# Hospital-Based Palliative and End-of-Life Care in the COVID-19 Pandemic: A Scoping Review

**DOI:** 10.1177/10499091211057049

**Published:** 2021-12-11

**Authors:** Michael Connolly, Mary Bell, Fiona Lawler, Fiona Timmins, Mary Ryder

**Affiliations:** 1 Clinical Nursing, School of Nursing, Midwifery & Health Systems, 8797 University College Dublin, Ireland; 2 8870 Our Lady’s Hospice & Care Services, Dublin, Ireland; 3 Nursing and Head of School, School of Nursing, Midwifery & Health Systems, 8797 University College Dublin, Dublin, Ireland; 4 School of Nursing, Midwifery & Health Systems, 8797 University College Dublin, Dublin, Ireland

## Abstract

**Aim**: To identify the nature of the evidence reporting hospital-based palliative and end-of-life care during the COVID-19 pandemic. **Background**: The COVID-19 pandemic has seen an increase in the numbers of seriously ill people being cared for across all health services worldwide. Due to the rapid progression of severe symptoms, the majority of staff working in hospitals and other healthcare centres were providing end-of-life care. Little is known about the level of hospital-based palliative care service provided during the COVID-19 pandemic, particularly during surges in admission rates with an increased number of deaths accruing. **Methods:** A scoping review was conducted to search and select potential studies. The scoping review was guided by the framework of Arskey and O’Malley and advanced by the use of the methodology of the Joanna Briggs Institute. **Results:** Eighteen studies published between March 2020 and July 2021 were identified. Three broad categories included overall management strategy and logistics, symptom prevalence and management of patients with COVID-19 and end-of-life care needs within the current pandemic. **Conclusions:** This review highlights increased awareness and knowledge of palliative and end-of-life care provided in hospitals. The review also highlights the response of hospital-based palliative care teams to an evolving crisis, within the context of developed health systems under sustained and overwhelming pressure. **Implications:** Newly established clinical links and referral pathways developed during the initial COVID-19 surge between hospital-based palliative care and other healthcare teams, be continued and further enhanced. Understanding of the role of specialist palliative care providers in supporting palliative and end-of-life care within the hospital setting needs further investigation.

## Introduction

The COVID-19 pandemic has seen a significant increase in the numbers of seriously ill people being cared for across all health services worldwide. Given the rapid progression of severe symptoms associated with a diagnosis of COVID-19, end-of-life care became the everyday work of the majority of staff working in hospitals and other health care centres during this time.

Palliative care is provided across a range of settings including hospital, community and in designated palliative care units or hospices. To date, hospital-based palliative care services provide a consultation service to assist in the management of complex symptoms and provide end-of-life care, and in some countries provide a link between hospital and the community. Little is known about the level of hospital-based palliative care service provision in the context of the COVID-19 pandemic, particularly when surges in admissions and deaths occurred. While it is reasonable to assume that palliative care services would be called upon to assist with complex symptom management and end-of-life care for those suffering with COVID-19, the benefit of specialised palliative care has not been visible within such an extreme environment to date or reported on.

### Aim of the Review

The aim of this scoping review was to evaluate the evidence about the use of palliative and end-of-life care during the COVID-19 pandemic in order to consider the gaps in knowledge and issues that require further investigation.

## Methods

A scoping review was conducted to identify and map the available evidence as well as to consider what future research may be needed.^1,2^ While this scoping study was conducted iteratively, Arksey and O’Malley^3^ methodological framework enhanced by Levac et al^4^ and formally adopted and developed further by the Joanna Briggs Institute^5^ was applied and Preferred Reporting Items for Systematic Reviews and Meta-Analyses (PRISMA)-ScR standards were used.^6^

### Stage 1- Identifying the Research Question

The research question was to scope the nature of the evidence concerning hospital-based palliative and end-of-life care in the COVID-19 pandemic. Key stakeholders from general hospital/tertiary care services helped to inform the research question and search strategy.

### Stage 2-Identifying the Relevant Studies

In collaboration with our specialist librarian and key stakeholders, a comprehensive search strategy was developed. Given the novel emergence of the COVID-19 pandemic, the focus of the literature search was restricted from March 2020 to July 2021.

The inclusion and exclusion criteria were refined iteratively among the team and after performing the search. The recommended Population, Concept and Context pneumonic for scoping reviews was used to structure the eligibility criteria^5,7^ (Table 1). The focus was on hospital-based palliative care during the COVID-19 pandemic. There were no restrictions to design or control. However, the search was limited to articles published in English. The inclusion criteria were further amended to specifically include the term hospital-based palliative care. Exclusion criteria included all papers that addressed non–hospital-based palliative care in a non-hospital setting during the COVID-19 pandemic and any papers that discussed related sub elements of palliative care with no reference to the COVID-19 pandemic.

**Table 1.** Inclusion and Exclusion Criteria.

PCC category	Inclusion criteria	Exclusion criteria
Population	Hospital-based palliative care COVID-19 pandemic patients, staff and relatives	Non–hospital-based palliative care in non-hospital COVID-19 pandemic
Concept	Palliative care	Not hospital based
	End-of-life care	COVID-19 pandemic not mentioned
Context	COVID-19 pandemic	Palliative care not mentioned
	Hospital based	

Database searches were undertaken in PUBMED, PsychINFO via ProQuest and CINAHL Complete. The search terms were related to hospital-based palliative care and COVID-19 and were searched as keywords as well as subject headings. For example, for hospital based, search terms used included acute setting, acute ward, inpatient, ward setting, acute care and ward. While the purpose of the searches was to identify the breadth of literature,^8^ as a consequence of limited researcher resource and time restrictions, other research techniques such as exploring the grey literature or reference checking of identified articles was not conducted.

The specialist librarian in the team stored the retrieved records on EndNote X9. Subsequently, Covidence, a web-based literature review software platform, was used to assist the screening and selection of records.

### Stage 3-Study Selection

**Figure 1.**
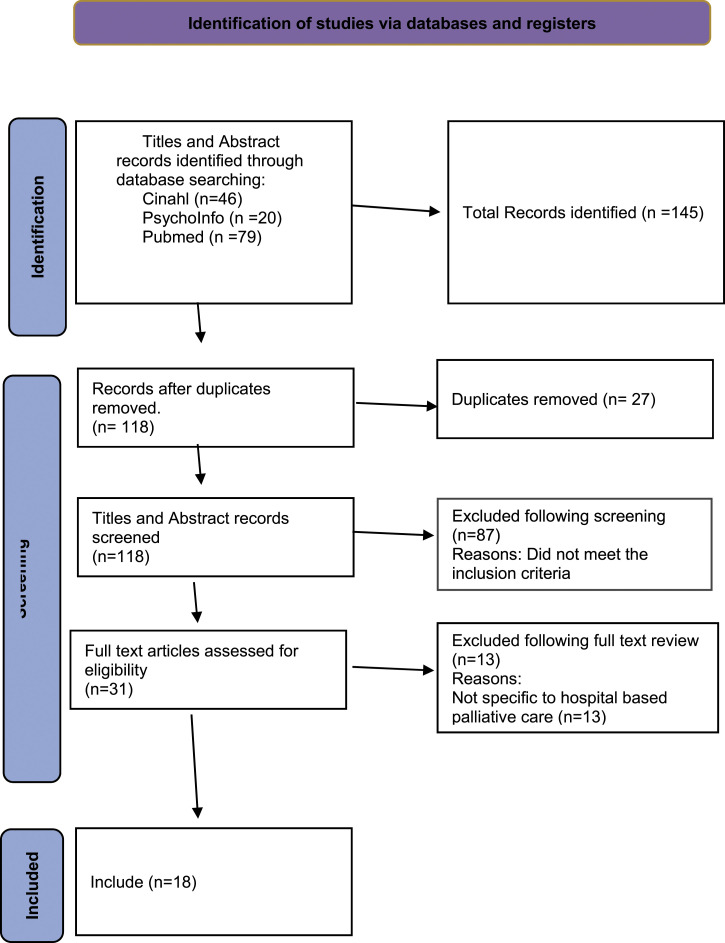
PRISMA statement.^9^

MC and MB screened all the titles and abstracts and undertook full-text review of the potentially relevant studies against the inclusion criteria. Any disagreements were discussed and resolved by consensus. The results are reported in the PRISMA flowchart^9^ (Figure 1).

### Stage 4- Charting the Data

MB undertook data extraction and initially drafted a charting table to reflect the relevant characteristics of the data. Charting the data became an iterative process, given the ongoing review and discussion with MC. It was further refined, reviewed and synthesised in accordance with Joanna Briggs Institute guidelines.^5^ Finally, the following agreed characteristics of the data were charted including author, year, country, journal, aim, population, methodology and key findings (Table 2).

**Table 2.** Data Extraction.

Author, Year	Country	Journal	Aim	Population	Methodology	Key findings
Alderman et al,2020^10^	UK	Palliative Medicine	To assess the utility of the effectiveness of standardised end-of-life care treatment algorithms in dying patients with COVID-19	61 patients	Audit-data extraction from end-of-life care plan	• Patients dying from COVID-19 experience similar end-of-life problems to other groups of patients
						• COVID-19 patients respond to standard interventions for these end-of-life problems
						• Cumulative number of patients experiencing shortness of breath, agitation and audible respiratory secretions increased over the last 72 hours of life-most patients symptom free at death
Anneser, 2020^11^	Germany	Palliative and Supportive Care	To identify the challenges and difficulties while caring for COVID-19 positive palliative patients in a non-ICU setting	1 patient	Case study	• Patient expressed distress at having no visitors
						• Patient was profoundly lonely
Arya et al, 2020^12^	Canada	CMAJ	To review the challenges involved in providing palliative care in a pandemic	N/A	Discussion	• In a pandemic, patient autonomy for end-of-life choices may be severely restricted due to public health guidelines and resource
						• Suggested Triage tool for referral to specialist PC
Chidiac et al,2020^13^	UK	Palliative Medicine	To evaluate the impact of COVID-19 on symptoms, clinical characteristics and outcomes for patients referred to a hospital-based palliative care service in a district general hospital	Compared 60 patients with COVID to 61 patients without COVID	Retrospective service evaluation. Data were extracted from the electronic patient records	• Lower comorbidity scores, poorer performance status + shorter time from referral to death for patients with COVID-19
						• Breathlessness, drowsiness, agitation, fever and use of s/c infusions had higher prevalence during COVID-19
						• BAME patients referred later to PC during COVID-19
						• Women from ethnic minority group referred later
						• Many patients referred for symptom control instead of terminal care-Need education on recognising dying for HCP
Cook et al,2020^14^	Canada	Annals of Internal Medicine	To understand clinician perspectives on adaptations to end-of-life care for dying patients and their families during the pandemic	45 dying patients, 45 relatives, 45 clinicians	Mixed methods embedded study	• Clinicians gathered 236 final wishes from patients and family-234 wishes were implemented
						• Clinicians: bigger effort to learn about pts; conducted acts of compassion; took on advocacy roles as the family not there due to restrictions; set up new ways of connecting with pts and relatives; prevent unmarked deaths
						• Clinicians reported moral distress with changing visiting policies
						• The need for clinicians to be aware of what they say and how they say it
						• IPC policies have implications for professional well-being-need to have feasible, context specific supports for frontline staff
Fausto et al,2020^15^	USA	Journal of Pain and Symptom Management	To implement a palliative care response plan for a multihospital health care system that incorporates conventional capacity, contingency capacity and crisis capacity	N/A	Development of a strategy document	• Document that outlines the high quality PC needed under conventional capacity, contingency capacity and crisis capacity
						• 5 considerations
						• Redeployment of specialist PC staff
						• Use PPE only when absolutely necessary
						• PC support outside routine hours
						• Routine PC consultation triaged and postponed where possible
						• Early goals of care are essential
						• 24 hr Phone support
						• In crisis capacity create an End-of-life care unit
Fiorentino et al, 2020^16^	USA	Palliative Medicine	To evaluate whether preadmission Palliative Performance Scale predicts mortality in hospitalised patients with COVID-19	334 patients	Retrospective observational cohort study of patients admitted with COVID-19	• Frailty was independently predictive of mortality in pts admitted with COVID-19
						• Low PPS found in older, mainly black with more comorbidities
						• Age and Charlson comorbidity index (CCI) did not independently predict mortality
						• Hospitalised pts = overall 31% mortality, 81% mortality for intubated pts
						• If clinicians understand mortality risk, can discuss prognosis and give appropriate PC to pts
Fox et al,2021^17^	Australia	Qualitative Social work	To discuss bereavement support and the facilitation of viewings as clinical areas in which hospital social work has been observed adapting practice creatively throughout the pandemic	N/A Social workers and their role	Discussion	• Change in practice by social workers during the pandemic to assist the bereavement process
						• Bereavement support
						• Facilitation of viewings of deceased patients
Gelfman et al,2020^18^	USA	Journal of Palliative Medicine	To describe the rapid expansion and creation of a new speciality care services across a health system to meet demands of the COVID-19 surge in New York city	1019 patients	Discussion	• Rapid expansion and creation of a new speciality PC service to address the pandemic demands
						• Increased staffing
						• 24 hour telephone service
						• Extended PC service to ED, ICU and hospital medical units
						• New tailored communication programme
						• COVID-specific electronic medical record template
						• PCU structural adaptation
Hetherington et al,2020^19^	UK	Palliative Medicine	To characterise the symptom profile, symptom management requirements and outcomes of hospitalised COVID-19 positive patients referred for palliative care and to contextualise PC demands from COVID-19 against a typical caseload	186 patients	Service evaluation based on a retrospective cohort review of patient records	• 186 pts referred to hospital PC with COVID-19
						• Most common comorbidities: Hypertension (31.2%); Diabetes Mellitus (28%); COPD (26.9%)
						• No referrals for PC from ICU
						• Dyspnoea and agitation most prevalent symptoms
						• COVID Pts referred to PC have shorter dying phase-2 days vs 5 days for pts with no COVID-higher death rate
						• Symptoms can be controlled in most cases with standard doses of opioids and benzodiazepines
						• 75% on SC infusions=effective in 78% of pts
Lopez et al, 2021^20^	USA	Journal of Pain and Symptom Management	To describe the characteristics, consultation demands, patients’ needs and outcomes of hospitalised patients with COVID-19 who received a palliative care evaluation	376 patients	Retrospective chart review	• Overall new consults significantly increased and especially in ICU
						• Median age = 78
						• 75% referrals to PC for goals of care, advanced care planning
						• 9.6% for symptoms
						• 7.1% had documented ACDs
						• 69.7% became DNR
						• Of all deaths, 55.5% in ICU and 87.2% were <65
						• Minority pts had disproportionate death rate
						• Overall consultation mortality significantly increased compared to pre-COVID
Lovell et al, 2020^21^	UK	Journal of Pain and Symptom Management	To describe the symptom burden, management response to treatment and outcomes for patients with COVID-19 referred to the palliative care teams in 2 large NHS hospital trusts	101 patients 76 admitted with covid 25 existing inpatients developed COVID	Descriptive Data extracted from case notes = Retrospective chart review?	• Patients spent a median (IQR] of 2 days under PC team and received 3 contacts
						• Most prevalent symptoms included: Breathlessness (n = 76), agitation (n = 43), drowsiness (n = 36), pain (n = 23)
						• 58 prescribed CSCI
						• n = 95 received ward based care and 6 in high dependency or ICU
						• Death (n =75)
						• Discharged back to team, home or hospice (n = 13)
						• Continued to receive inpatient PC (n = 13)
						• Infusions assessed as partially effective for 40/58, while 13 pts died before review
Moriyama et al, 2021^22^	USA	Journal of Pain and Symptom Management	To describe a palliative care population at 1 New York hospital system during the initial pandemic surge	678 patients =pre-COVID group	Cross-sectional, observational study.	• PC service volume surged from 678 (4% of total admissions) pre-Covid to 1071 (10% of total admissions during initial outbreak
						• PC teams completed 59% (n = 1081)increase in admissions compared to pre-Covid admissions
						• 64.9% (n = 695) of total PC pts tested positive for COVID

				Compares pre-Covid group (Jan 4th-Feb 28th, 2020) to during Covid group (Mar 5th-30th April)		
						• Patients with COVID had greater prevalence of
						• Obesity
						• Diabetes
					All data were extracted from charts	• ICU admissions (58.9% vs 33.9%; P<.01)
						• In-hospital mortality (57.4% vs 13.1%; P<.01)
						• Males (60.7% vs 48.6%, P<.01)
						• Latino patients (21.3% vs 13.3%,P<.01)
						• Increased odds of mortality in PC patients (odds ratio = 3.21,95% CI = 2.43-4.24) and those admitted to ICU (odds ratio = 1.45, 95%CI = 1.11-1.9)
						• Patients with COVID had lower rates of
						• End-stage organ disease
						• Cancers
Mumoli et al,2020^23^	Italy	International Journal of Infectious Disease	To describe the role of palliative care in the management of patients admitted with COVID-19	412 patients	Descriptive study recorded data regarding age, gender, length of stay, type of discharge	• 412 pts admitted to ward with COVID
						• Mean age = 69
						• 23.3% (n = 96) died in hospital
						• 4.6% (n = 19) came from nursing homes
						• Palliative Care Physician directly involved with 25.5% (n = 105) and conducted 236 consultations
						• Of the 105, 63% (n = 66) died
						• Reasons for consultations included: symptoms (54%) and end-of-life management (12%)
						• Prevalent symptom: Restlessness/agitation (41%), emotional issues (26%) such as anxiety, fear and demoralisation
						• Dyspnoea in 20% of cases
						• Patients discharged alive or transferred to other facilities-median hospital stay = 12 days
						• Average days in PC = 2.26
						• Palliative Care physician developed shared operational procedures in the following areas
						• Criteria for when COVID pts to start PC treatment
						• Shared decision-making process with other physicians
						• Managed anxiety and stress regarding intolerance to respiratory devices
						• Administered palliative sedation at end of life
						• Communication with patients and families
						• Chose medications and how to administer them
						• Protected the self-determination of patients
						• Defined 6 criteria for referral to PC
						• MDT +PC met daily to discuss each patient with COVID
Selman et al, 2021^24^	UK	Journal of Pain and Symptom Management	To review bereavement risk factors in COVID-19 and provide evidence-based recommendations for clinicians on how to support bereaved relatives	N/A Staff	Discussion paper with recommendations of the organisational and systemic approaches needed to mitigate the impact on staff	• Provides evidence based recommendations and table of resources to assuage poor bereavement outcomes before and after a patient’s death for relatives and support staff
						• Recommendations

						• For relatives
						• ACP
						• Communication-sensitive, regular, and informative
						• Enable family to say goodbye
						• Provide excellent symptom management +emotion+spiritual support
						• Provide/signpost bereavement services
						• For staff
						• Consistent leadership
						• Resources, guidance + training
						• Actively monitor frontline staff
						• Professional sources of support made available
						• Strategies to support teams day-to-day work
						• Several risk factors for poor bereavement outcomes in COVID-19
						• Dying in an ICU
						• Severe breathlessness
						• Patient isolation or restricted access
						• Significant patient and family emotional distress
						• Disruption to relatives’ social support networks
						• Risk of the impact of deaths on staff
						• Secondary or vicarious trauma
						• Moral injury
						• Depression
						• Anxiety
						• Post-traumatic distress
Sheehan et al, 2020^25^	USA	BMJ Supportive and Palliative Care	To examine the utilisation rates of palliative care consultation in critically ill patients with COVID-19 pneumonia admitted to 2 ICUs in New York city	151 ICU patients with confirmed COVID-19 pneumonia	Retrospective cohort study, data collected from the electronic health records	• Underutilisation of PC services in ICU
						• PC delivery-shared decision-making, ACP, symptom management mainly conducted using telemedicine
						• Identify patients at risk of poorer outcomes that would benefit from earlier PC consultation
						• Of 39% (n = 59) received inpatient PC consultation, 16 received a one-time telemedicine consultation, 39 received continued telemedicine and follow-up +MDT involvement and 4 patients initially received one-time telemedicine followed by continued telemedicine
						• 56.29% (n = 85/151) died-57.65% (n = 49/85) received PC during hospitalisation
						• Those who died and received PC were: older, higher CCI and needed mechanical ventilation
						• Of patients who died and did not receive PC were: younger, received no invasive ventilation support
						• Propensity matching: PC consultation was associated with similar length of stay to patients who did not receive PC services
Turner et al, 2020^26^	UK	Journal of Pain and Symptom Management	To present a case series from audit data collected from an acute hospital trust specifically looking at the palliative care patients with COVID-19 received in a ward setting	36 patients	An observational retrospective review using patients records	• Increased frailty and co-morbidities featured heavily with cohort of patients in this study
						• Median age 81
						• Dying from COVID-19 is different
						• Shorter average dying phase-38.25 hours vs 74 hours
						• 3 different phenotypes of dying
						• Fulminant COVID-19
						• Longer illness and slower death
						• Long illness, stability and rapid death
Xu et al,^27^ 2021	USA	Journal of Palliative Medicine	To describe the dynamic palliative care needs during a public hospital’s COVID-19 surge, including a process to utilise nonpalliative care trained volunteers to meet the increased demand for inpatient palliative care consults	12 volunteers joined PC team and saw 276 patients	Discussion paper	• Model to address surge
						• 12 volunteers including 6 psychiatrists, 1 paediatrician, 5 internal medicine and 1 nurse practitioner
						• One day training-shadowed a PC team member and given a manual
						• PC team saw all patients with multiple organ failure
						• Psychiatrists quickly used own skills in communication to support family and staff
						• PC specialists focussed on more complex symptom needs of particular patients
						• ICU staff too overwhelmed to request PC consults by phone or email
						• PC team grew by 35%
						• Changes to PC team structure to address surge
						• Majority of consults were for goals of care and family support
						• PC team supported frontline ICU staff who were working out of their comfort zone

### Stage 5-Collating, Summarising and Reporting the Results

The analysis of the extracted data incorporated 2 steps: the first being a descriptive numerical summary of the relevant characteristics of the data and the second was a descriptive content analysis that captured the overall main categories of literature that addressed the research question for this scoping study.

## Results

### Selection of Studies

The search initially yielded 145 articles (Figure 1). With 27 duplicates removed and when title and abstracts were reviewed another 87 were excluded as they did not meet the inclusion criteria. The remaining 31 studies were assessed for full-text eligibility resulting in a further 13 being excluded as these were not specific to hospital-based palliative care. The remaining 18 underwent full-text review and were included in the study.

### Description of Studies

Overall, this review process gleaned a broad variety of articles that focussed on different aspects of data capturing palliative and end-of-life care during the COVID-19 pandemic within hospitals. It was noteworthy that 14 out of the 18 articles were published in dedicated palliative care journals. Of the 18 articles, 7 were from USA, 6 from UK, 2 from Canada and 1 each from Australia, Germany and Italy. This result highlights an absence of papers in English from South America, Asia, or Africa where some countries had some of the highest rates of COVID-19 and consequent deaths.^28^

The predominant methodological approach adopted was retrospective patient chart review or care plan analysis reported in 9 articles.^10,13,19-23,25,26^ Of 6 discussion articles identified, 2 examined bereavement risk^17,24^ 1 presented the challenges experienced in delivering palliative services during a pandemic^12^ and 3 discussed the implementation of a strategy or plan that was developed in their hospital to address the impact of the surge in palliative care patients.^15,18,27^ One article presented a patient case study of the challenges and difficulties that were identified in a non-ICU setting.^11^ Fiorentino et al. (2020)^16^ conducted a retrospective observational study using the Palliative Performance Scale (PPS) to predict mortality in 334 hospitalised patients with COVID-19. Cook et al. (2021)^14^ used mixed methodology to investigate clinicians’ perspectives on the adaptation to end-of-life care for dying patients and their families during the pandemic.

The sample sizes used in the 9 chart reviews ranged from 36^26^ to 1071 patients.^22^ Two of these studies compared palliative care services needs of 2 groups of patients. Chidiac et al. (2020)^13^ compared 60 patients with COVID-19 and another 61 without, to evaluate the impact of COVID-19 on symptoms, clinical characteristics and outcomes for patients in a hospital-based palliative care service. Moriyama et al. (2021)^22^ compared 678 patients, the pre-COVID group, to 1071, the COVID-19 group regarding their demographics, comorbidities and rates of mortality.

Following an analysis of the extracted data, 3 broad categories were identified reflecting the predominant perspectives of addressing palliative and end-of-life care needs during a pandemic within hospital settings. These included overall management strategy and logistics, symptom prevalence and management of patients with COVID-19 and end-of-life care needs/consultation demands within the current pandemic.

### Overall Management Strategy and Logistics

The overall strategies and logistics adopted to address the surge in demand for palliative care was reported by 3 articles from the USA and 1 from Canada.^12,15,18,27^ Each outlined a strategic plan that was adopted and focussed primarily on how to increase the numbers of palliative care staff as well as optimising the use of existing palliative care specialists. Some novel ways of addressing this problem were reported. For example, Xu et al. (2021)^27^ described how 12 volunteer clinicians (11 physicians and 1 nurse practitioner) each shadowed a palliative care team member for 1 day, given a manual with communication guidance, received targeted training on advanced care planning and was then embedded within a team led by a palliative care provider. Consequently, it emerged that 6 of these physicians were psychiatrists and could quickly use their own communication skills to support family and staff. What was also apparent from these papers was the necessity for very quick training of staff not specialised in palliative care that worked in key areas such as ED or ICU to enhance their frontline skills via new tailored communication trainings.^18^ Fausto et al. (2020)^15^ reported that all teams in ED, ICU and acute care services were provided with clinician discussion tools and access to consultation with the specialist palliative care team for assistance with complex communication. These frontline staff were provided with resources that included an informed assent strategy for discussing do-not-resuscitate orders as well as resources for COVID-19 ready communication skills. Arya et al. (2020)^12^ presented 2 short scripts capturing the suggested language that clinicians should use when providing support to a patient or family member who was denied access to intensive care due to resource scarcity and when discussing treatment when a patient is unlikely to survive a critical illness but could include life-sustaining therapies if needed.

Besides redeployment of non-specialist staff into the palliative care team, cognisance of the most efficient and effective use of specialist palliative care clinicians were also evident. A triage tool for referral to specialist palliative care was adopted as part of the mass casualty critical care framework by Arya et al. (2020),^12^ while Fausto et al. (2020)^15^ described how routine palliative care consultations were triaged and postponed when possible. A palliative care specialist was also embedded in the ED and ICU to address and screen high volumes of patients, to coach staff and to assist with goals of care.^15^ Xu et al. (2021)^27^ reported how specialist palliative care teams expanded their role and saw every patient with multi-organ failure in ICU and took over the communication with families. They also acknowledged that due to the urgency of the situation and the swift nature of the implementation of their interventions to address the COVID-19, outcomes could not be effectively measured at that time.^27^

New services were also developed to address the many challenges that arose due to surges in COVID-19 admission. A 24-hour/afterhours telephone service was established to provide additional palliative care capacity to primary care teams in relation to symptom guidance and coaching^15^ and to patient’s families.^18^ Specific end-of-life care units for patients dying with COVID-19 staffed by palliative care clinicians and advanced practice specialists were created^15^ or modified^18^ to maximise patient comfort and staff safety.

### Symptom Prevalence and Management of Patients With COVID-19

Five articles reported on symptom prevalence and descriptive statistics of patients admitted to hospital during with COVID-19.^13,16,19,21,23^ Dyspnoea and agitation were the 2 most prevalent symptom reported by 3 studies.^13,19,21^ By contrast, an Italian study reported that 41% of patients’ predominant symptom was restlessness/agitation, 26% was emotional issues but only 20% presented with dyspnoea as the most prevalent symptom.^23^

Comparisons were made between referral patterns for those admitted with COVID-19 and those referred to specialist palliative eservices pre-COVID-19. There were some notable observations. The time from referral to palliative care to death was shorter^13,19,21,23^; specifically, 2 days for patients with COVID vs 5 days for those without. Fiorentino et al. (2020)^16^ used the PPS and found that frailty was an independent predictor of mortality. In addition, patients with COVID-19 had a lower PPS found in older patients and predominantly black patients with comorbidities. The most common comorbidities for patients with COVID-19 were hypertension, diabetes mellitus and COPD.^19^ However, age and the Charlson Comorbidity Index did not predict mortality in the Fiorentino et al.’s (2020)^16^ study.

### End-Of-Life Care Needs/Consultation

Both Lopez et al. (2021)^20^ and Sheehan et al. (2020)^25^ examined the utilisation rates and characteristics of consultations to palliative care during the initial wave of the pandemic. Not surprisingly, new consults increased significantly. Three quarters (75%) of referrals to palliative care were for goals of care and advanced care planning while less than 10% were for symptom management.^20^ Both of these studies showed that less than ten percent had documented Advance Care Directives despite the sample population age being older. It is noteworthy that Chidiac et al. (2020)^13^ reported that 40% (n = 24) of patients who were referred to palliative care for symptom control were evidently in the dying phase of COVID-19 illness. In addition, Sheehan et al.^25^ study focussed on patients in the ICU setting and found that there was an underutilisation of palliative care services.

PC consultations varied in how they were managed. Sheehan et al. (2020)^25^ indicated that 39% (n = 59) received inpatient PC consultation, 16 received a one-time telemedicine consultation, 39 received continued telemedicine and follow-up and MDT involvement and 4 patients initially received one-time telemedicine followed by continued telemedicine.

It is worth noting that 1 study found that patients dying from COVID-19 experience similar end-of-life care needs and problems to other groups of patients as well as responding to standard interventions for those end-of-life problems.^10^

### Identified Impact

It is clear that the infection, prevention and control restrictions during the COVID-19 pandemic led to a number of adverse impacts to patients and staff in a number of articles. A German case study reported how 1 patient expressed distress at having no visitors and was profoundly lonely.^11^ In order to respond to the impact of visiting restrictions, clinicians in a Canadian study took on advocacy roles for patients.^14^ For example, these clinicians ascertained the final wishes of patients and their families and implemented them, setup new ways of connecting patients with their relatives and actively tried to ensure that no patient death was unmarked. Social workers in Australia assisted the bereavement process for families by changing their practice using memory making as a tool to support families through letters, photos and hand-drawn pictures and music.^17^ Cook et al. (2021)^14^ also reported how the burden on clinicians associated with visitor restrictions led to clinicians reporting moral distress. Indeed, Selman et al. (2020)^24^ identified several risk factors for poor bereavement outcomes for relatives including dying in the ICU, severe breathlessness, patient isolation and significant patient and family emotional distress. The impact of these deaths on staff led to self-reported moral distress/injury, depression, anxiety and post-traumatic distress.

Uniquely, Turner et al. (2020)^26^ from a chart review observed that there were 3 different ways of dying from COVID-19 labelled as fulminant COVID-19, longer illness and slower death or long illness, stability and rapid death. However, many of these studies reported a shorter average dying phase for those with COVID-19 of about 38.25 hours compared to 74 hours with no COVID-19.

## Discussion

Eighteen studies were identified for inclusion which used a variety of methods to detail varying different experiences of hospital-based palliative teams’ response to COVID-19.

The studies articulated a comprehensive response to COVID-19 provided by hospital-based palliative care teams, including interventions to manage symptoms, providing supportive care to patients, families and staff and a novel solution to providing palliative and end-of-life care by appropriately triaging those with worsening COVID-19 symptoms. This process of triage saw palliative and end-of-life care being centrally located in the planning and provision of care to patients with COVID-19.

While palliative and end-of-life care have received attention in the past, this review clearly articulates the immediate and ongoing response of hospital-based palliative care to a continually emerging health care emergency which resulted in a significant increase of patients needing specialist palliative and end-of-life care. It also highlights the role that hospital-based palliative care played in supporting staff caring for the dying, including staff who have not experienced this type of care provision before, while also recognising the large numbers of dying patients being cared for. It is evident from this review that palliative care became more visible during surges in admissions due to COVID-19.

This review highlights the response of hospital-based palliative care in the context of a pandemic. It demonstrates the myriad ways in which hospital-based palliative care responded to the pandemic, for example, symptom management advice, communication with patients and families about change in focus of care and end-of-life care planning. This review adhered to the relevant reporting framework and was rigorous in its methodology.

The majority of studies included were small single centred with small population samples. Only 6 of the studies had a sample population greater than 100. While a comprehensive research strategy was conducted, the studies included are all English language, so the experience of hospital-based palliative care not reported in English could not have been included.

## Conclusion

This review has highlighted the increased awareness and knowledge of palliative and end-of-life care provided in hospitals. The COVID-19 pandemic and subsequent increased admission to hospitals has led to increased referral rates to these services, which in turn has the potential to improve patients and family care and assist with greater interaction and consultation with specialist palliative care in the future. The review has also highlighted the rapid response of hospital-based palliative care teams to an evolving crisis, within the context of developed health systems under sustained and overwhelming pressure.

### Implications for Practice

It is imperative that newly established clinical links and referral pathways developed during the initial COVID-19 surge between hospital-based palliative care and other health care teams be continued and further enhanced to ensure that patients’ palliative and end of life remains a focus for those with life-limiting illnesses and be included as part of their planned care.

Understanding of the role of specialist palliative care providers in supporting palliative and end-of-life care within the hospital setting needs investigation.
